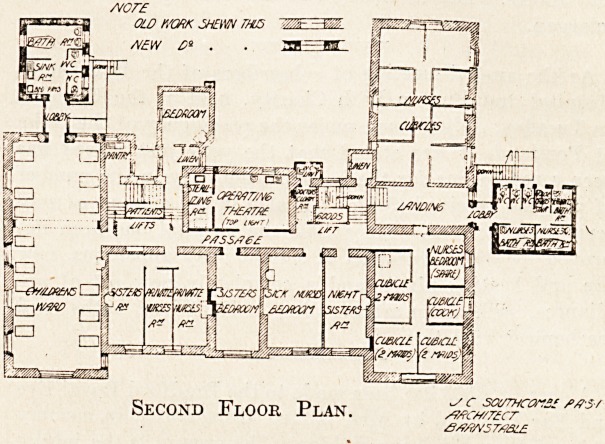# Additions to the North Devon Infirmary at Barnstaple

**Published:** 1907-08-31

**Authors:** 


					ADDITIONS TO THE NORTH DEVON INFIRMARY AT BARNSTAPLE.
This infirmary was originally built about eighty years
ago; and it is not too much to say that since that time hos-
pital construction has been almost completely revolutionised.
It is therefore not to be wondered at that much of the old
building was greatly behind the requirements of the present
day; and more especially was this the case as regards the
sanitary arrangements and the cross-ventilation of the
wards. The committee have spent about ?4,650 in new
work and in rearranging wards, pulling down partitions and
putting in more windows. In addition to this sum about
?300 will be spent on external staircases.
The infirmary, as altered, was formally reopened in April
last by the Countess Fortescue, and we print herewith the
plans of the building as now in use. To bring
an old hospital up to the modern standard in all
its parts would be practically tantamount to
erecting a new one, and it is but seldom that
funds are at hand for this purpose. The managers
of the Barnstaple Infirmary may, however, fairly
claim to have wiped out many of the most serious
"blots; and that it is not better than it is now is
not so much their fault as that of the original
plan. This fact should be borne in mind.
The basement contains the kitchen and other
portions of the administrative department, and
also at the north-west side the patients' entrance,
waiting-room, women's surgery, men's surgery,
and eye-room. From this, casualty-department
patients can be taken on a trolley to the lift, and
so to the operating theatre, without difficulty.
The ground floor has in its south-east front the entrance
hall, which opens into a corridor, on one side of which are
the medical officers' rooms and board-room; and on the
other side are the serving-rooms, waiting-rooms, staircases,
and a passage leading to the nurses' duty-room, the isolation
ward for two beds, and the entrance lobby to that ward.
This part of the hospital is well arranged. At the west end
of the corridor is the door leading to the men's ward. This
ward contains ten beds. It is about 55 feet long and 20 feet
wide. Each bed has about 11 feet of wall space, 110 square
feet of floor space, and, assuming a ceiling height of 12 feet,
about 1,320 cubic feet of air space. This is not excessive;
but one fault of the ward is its width, which should not be
less than 24 feet, and another is the number and disposition
of the windows. Of the ten beds only three have windows
on both sides, and three others have no windows at all.
There is, however, a large window in the south-
east end of the ward, and two smaller ones at the
other end, which will materially help the ventila-
tion ; nevertheless, beds should not be placed against a dead
wall. The new sanitary annexe projects from the north-
west end. It is properly and efficiently cut off from the
main building by a lobby, and it contains bathroom, sinks,
and closets. There is a pantry attached to this ward, but
neither duty-room nor single-bedded ward. At the south-
east end of the main corridor are the matron's rooms, the
linen room, the chapel, and a new annexe, containing bath-
room, storeroom, and closet.
The first floor contains another men's ward for ten beds,
the arrangements being similar to those of the ground floor
This infirmary was originally built about eighty years the medical officers' rooms and board-room; and on the
ago; and it is not too much to say that since that time hos- other side are the serving-rooms, waiting-rooms, staircases,
pital construction has been almost completely revolutionised. and a passage leading to the nurses duty-room, the isolation
It is therefore not to be wondered at that much of the old NORTH DEVON INFIRMARY BflRNSTflPiE
building was greatly behind the requirements of the present aoncnspn fit TFDPT/DM^
day; and more especially was this the case as regards the
sanitary arrangements and the cross-ventilation of the
wards. The committee have spent about ?4,650 in new
work and in rearranging wards, pulling down partitions and
putting in more windows. In addition to this sum about
?300 will be spent on external staircases.
The infirmary, as altered, was formally reopened in April
last by the Countess Fortescue, and we print herewith the
plans of the building as now in use. To bring
an old hospital up to the modern standard in all
its parts would be practically tantamount to
erecting a new one, and it is but seldom that
funds are at hand for this purpose. The managers
of the Barnstaple Infirmary may, however, fairly
claim to have wiped out many of the most serious
blots; and that it is not better than it is now is
not so much their fault as that of the original
plan. This fact should be borne in mind.
The basement contains the kitchen and other
portions of the administrative department, and
also at the north-west side the patients' entrance,
waiting-room, women's surgery, men's surgery,
and eye-room. From this, casualty-department
patients can be taken on a trolley to the lift, and
so to the operating theatre, without difficulty. B/is?flENT FJ./7A/
The ground floor has in its south-east front the entrance
hall, which opens into a corridor, on one side of which are
>vor?
OW WORK SHCWN w
^ c samxnx Mst
&/POW/D /ZOOff PL/7A/
ward for two beds, and the entrance lobby to that ward.
This part of the hospital is well arranged. At the west end
of the corridor is the door leading to the men's ward. This
ward contains ten beds. It is about 55 feet long and 20 feet
wide. Each bed has about 11 feet of wall space, 110 square
feet of floor space, and, assuming a ceiling height of 12 feet,
about 1,320 cubic feet of air space. This is not excessive;
but one fault of the ward is its width, which should not be
less than 24 feet, and another is the number and disposition
of the windows. Of the ten beds only three have windows
on both sides, and three others have no windows at all.
There is, however, a large window in the south-
east end of the ward, and two smaller ones at the
other end, which will materially help the ventila-
tion ; nevertheless, beds should not be placed against a dead
wall. The new sanitary annexe projects from the north-
west end. It is properly and efficiently cut off from the
main building by a lobby, and it contains bathroom, sinks,
and closets. There is a pantry attached to this ward, but
neither duty-room nor single-bedded ward. At the south-
east end of the main corridor are the matron's rooms, the
linen room, the chapel, and a new annexe, containing bath-
room, storeroom, and closet.
The first floor contains another men's ward for ten beds,
the arrangements being similar to those of the ground floor
August 31, 1907. THE HOSPITAL. 591
ward. The space over the board-room is given up to an
emergency ward for four beds, and that over the serving-
room and waiting-room to a ward kitchen, while the night
nurses find their bedrooms over the isolation ward nurses'
duty-room.
The women's ward is placed over the chapel and matron's
rooms. It is about 80-feet long and 20 feet wide, and
contains fourteen beds, each bed having about 115 square
feet of floor space. Two beds'are supplied with a window
on both sides, nine on one side only, and three on neither
side. The sanitary annexe projects from the east side of
the ward, and is similarly constructed to that already de-
scribed, and is cut off from the ward by a ventilating lobby.
The second floor has the children's ward over the men's
ward. It contains fourteen cots.
The operating theatre is correctly placed to the north.
It has a sterilising-room attached to it, and is lighted by a
north window and a roof light. The floor is of terrazzo, and
coves take the place of corners, the walls being lined with
white glazed tiles. The rest of the second floor is given up
to bedrooms and cubicles for the staff.
The mortuary has been enlarged and a post-mortem room
added. Other improvements are a new larder, new heating
arrangements, and a hot-water installation.
Mr. J. C. Southcombe was the honorary architect, and
the work was carried out under the superintendence of Mr.
Spencer Edwards. The contractor for the building was
Mr. William Slee. Messrs Williams and Company and
Messrs. Parkin and Sanders did the engineering parts of
the new work.
1^
1 |_ re
m**" "S.
* flPVe /><
First Floor Plan.
/vor?
OLD K'WK SHEW MVS
^ C SC(/THCai*f S/7-5/
Second Floor Plan. /v/pc/v/t^ct
0/7/?/V5T/?at?

				

## Figures and Tables

**Figure f1:**
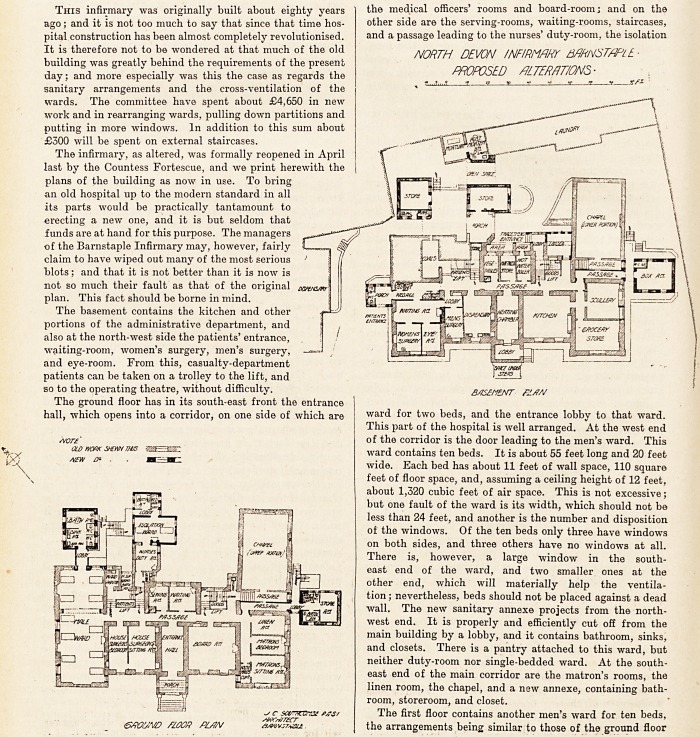


**Figure f2:**
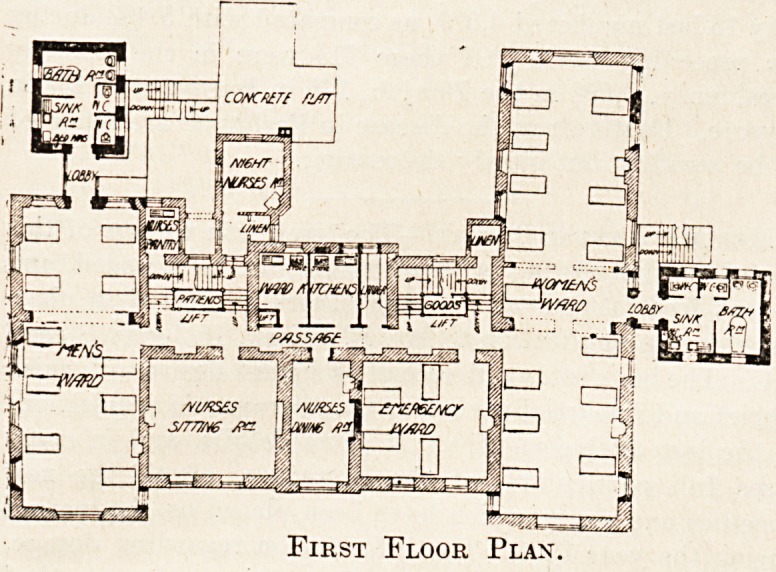


**Figure f3:**